# MetArea: a software package for analysis of the mutually exclusive occurrence in pairs of motifs of transcription factor binding sites based on ChIP-seq data

**DOI:** 10.18699/vjgb-24-90

**Published:** 2024-12

**Authors:** V.G. Levitsky, A.V. Tsukanov, T.I. Merkulova

**Affiliations:** Institute of Cytology and Genetics of the Siberian Branch of the Russian Academy of Sciences, Novosibirsk, Russia Novosibirsk State University, Novosibirsk, Russia; Institute of Cytology and Genetics of the Siberian Branch of the Russian Academy of Sciences, Novosibirsk, Russia; Institute of Cytology and Genetics of the Siberian Branch of the Russian Academy of Sciences, Novosibirsk, Russia Novosibirsk State University, Novosibirsk, Russia

**Keywords:** de novo motif search, PR curve, area under curve, structural variants of transcription factor binding site motifs, cooperative action of transcription factors, de novo поиск мотивов, кривая PR, площадь под кривой, структурные варианты мотивов сайтов связывания транскрипционных факторов, кооперативное действие транскрипционных факторов

## Abstract

ChIP-seq technology, which is based on chromatin immunoprecipitation (ChIP), allows mapping a set of genomic loci (peaks) containing binding sites (BS) for the investigated (target) transcription factor (TF). A TF may recognize several structurally different BS motifs. The multiprotein complex mapped in a ChIP-seq experiment includes target and other “partner” TFs linked by protein-protein interactions. Not all these TFs bind to DNA directly. Therefore, both target and partner TFs recognize enriched BS motifs in peaks. A de novo search approach is used to search for enriched TF BS motifs in ChIP-seq data. For a pair of enriched BS motifs of TFs, the co-occurrence or mutually exclusive occurrence can be detected from a set of peaks: the co-occurrence reflects a more frequent occurrence of two motifs in the same peaks, while the mutually exclusive means their more frequent detection in different peaks. We propose the MetArea software package to identify pairs of TF BS motifs with the mutually exclusive occurrence in ChIP-seq data. MetArea was designed to predict the structural diversity of BS motifs of the same TFs, and the functional relation of BS motifs of different TFs. The functional relation of the motifs of the two distinct TFs presumes that they are interchangeable as part of a multiprotein complex that uses the BS of these TFs to bind directly to DNA in different peaks. MetArea calculates the estimates of recognition performance pAUPRC (partial area under the Precision–Recall curve) for each of the two input single motifs, identifies the “joint” motif, and computes the performance for it too. The goal of the analysis is to find pairs of single motifs A and B for which the accuracy of the joint A&B motif is higher than those of both single motifs.

## Abstract (ru)

Технология ChIP-seq, основанная на иммунопреципитации хроматина (ChIP), позволяет картировать набор геномных локусов (пиков), содержащих сайты связывания (СС) для исследуемого (целевого) транскрипционного фактора (ТФ). ТФ может распознавать несколько структурно различных мотивов СС. Мультибелковый комплекс, картируемый в эксперименте ChIP-seq, включает целевой и другие «партнерские» ТФ, связанные белок-белковыми взаимодействиями. Не все из этих ТФ связываются с ДНК напрямую. Поэтому и целевой, и партнерские ТФ распознают обогащенные мотивы СС в пиках. Для поиска обогащенных мотивов по данным ChIP-seq применяется подход de novo поиска. Для пары обогащенных мотивов СС ТФ в наборе пиков может быть обнаружена совместная или взаимоисключающая встречаемость: совместная отражает более частое нахождение двух мотивов СС ТФ в одних пиках, а взаимоисключающая – в разных пиках. Мы предлагаем программный комплекс (ПК) MetArea для выявления пар мотивов СС ТФ со взаимоисключающей встречаемостью по данным ChIP-seq. ПК MetArea предназначен для предсказания структурного разнообразия мотивов СС одного ТФ и функциональной связи мотивов СС разных ТФ. Функциональная связь мотивов двух разных ТФ предполагает, что они взаимозаменяемы в составе мультибелкового комплекса, который использует СС этих ТФ для прямого связывания с ДНК в различных пиках. ПК MetArea рассчитывает оценки точности распознавания pAUPRC (частичная площадь под кривой Precision–Recall) для каждого из двух входных одиночных мотивов, определяет их «объединенный» мотив и оценивает точность для него. Целью анализа является поиск пар одиночных мотивов A и B, для которых точность объединенного мотива A&B выше точностей обоих одиночных мотивов.

## Introduction

Transcription factors (TFs) are proteins that have the ability to specifically bind DNA and thereby regulate gene transcription. About 1,600 human proteins are TFs (Lambert et al., 2018). TF binding sites (BSs) in eukaryotic genomic DNA are short regions, typically 6 to 20 base pairs (bp) in length (Vorontsov et al., 2024). TFs are usually able to bind not to a single DNA sequence, but to many similar ones. The TF BS motif in DNA is a general representation of the available diversity of such similar sequences (D’haeseleer, 2006). It is very difficult to establish clear patterns that determine the affinity of nucleotide sequences of genomic DNA to TFs. Only a few nucleotide positions are at least moderately conserved in TF BS motifs, i. e. they are unchanged in most natural BSs. Typically, the number of such positions is much less than a half of a motif length. The diversity of TF BS motifs in vivo is still very poorly studied because of the great variety of TF binding mechanisms to DNA. They include, in addition to direct binding, binding by other TFs or through them as intermediaries, use of the spatial structure of DNA within the nucleosome for binding, etc. (Morgunova, Taipale, 2017; Levitsky et al., 2020; Zeitlinger, 2020).

The most popular model of TF BS motifs is the traditional positional weight matrix (PWM) (Wasserman, Sandelin, 2004; Tognon et al., 2023). The PWM estimates the affinity of a site as the sum of the contributions (weights) of all its positions, where the weight of each position is defined by its nucleotide type. Alternative motif models are able to complement the predictions of the PWM model (Levitsky et al., 2007; Siebert, Söding, 2016; Tsukanov et al., 2022), i. e. to predict TF BSs in such genomic loci where the PWM model does not. The common difference between all alternative motif models and the traditional PWM model is the assessment of site affinity through the contribution of nucleotide frequency dependences between different motif positions.

DNA-binding domains (DBDs) provide TFs the ability to interact with DNA. The structure of a TF’s DBD determines the variants of its BS motifs (Wingender, 2013; Lambert et al., 2018; Nagy G., Nagy L., 2020). Hierarchical classification of TFs based on the DBD structure in the TFClass database (Wingender, 2013; Wingender et al., 2013, 2015, 2018) defines classes of TFs based on their DBD structure. For example, the Hocomoco database (Vorontsov et al., 2024) annotates the BS motifs of 949 different human TFs. These TFs belong to 34 classes, but ten classes with at least ten TFs account for 858 TFs (more than 90 % of all 949 TFs), and the three largest classes, C2H2 zinc finger factors {2.3}, Homeo domain factors {3.1}, and Basic helix-loop-helix factors (bHLH) {1.2} include 373, 184, and 76 TFs, respectively. The alignment of TF DBD sequences defines families and subfamilies of TFs below the classes in the hierarchy.

TFs of eukaryotes interact with DNA in vivo as part of multiprotein complexes including several TFs. TFs in such complexes are called “partner TFs”, as there are protein-protein interactions between them. The common (cooperative) action of several TFs on the regulatory region of a gene is able to change the local environment of chromatin and regulate gene transcription (Morgunova, Taipale, 2017; Zeitlinger, 2020; Georgakopoulos-Soares et al., 2023). Many classes of TFs are characterized by the ability of TFs to bind to completely structurally different BSs (Rogers et al., 2019; Vorontsov et al., 2024). For example, TFs of the “Nuclear receptors with C4 zinc fingers {2.1}” class can bind as monomers and dimers. In the dimer case, the BS includes two half-sites; the spacer between them and the DNA strands of half-sites can vary. TFs of the “Basic leucine zipper factors (bZIP) {1.1}” class bind only as dimers, two half-sites are always located in the same DNA strand and the spacer is almost unchanged (Nagy G., Nagy L., 2020). Hereinafter, indices in curly brackets are labelled according to the TFClass database (Wingender et al., 2013, 2015, 2018). There are several types of DBDs of eukaryotic TFs that can function as dimers including pairs of closely related TFs (Amoutzias et al., 2008). TFs similar in DBD structure often recognize similar TF BS motifs (Lambert et al., 2018; Ambrosini et al., 2020), with the only clear exception to this rule being the BS motifs of TFs from the “C2H2 zinc finger factors {2.3}” class.

The identification of TF BSs in genomes has advanced significantly in the last 15 years with the advent of highthroughput massive sequencing methods, in particular, the experimental ChIP-seq technology. This technology gives for the target TF a set of genomic loci (peaks), usually several hundred bp in length, where the binding of the multiprotein complex of many TFs, including the target TF, has been experimentally mapped. Therefore, two types of peaks are responsible for direct and indirect binding of the target TF to genomic DNA. Direct binding means that the target TF is bound to DNA directly, and indirect binding means that the target TF is bound only by protein-protein interactions with one or more partner TFs, which in turn are bound to DNA directly. The presence of direct/indirect binding implies that the BS motifs of the target/partner TFs are enriched in the peaks, and the motifs of the target TFs are present only in part of the peaks. The term “enrichment” is used to reflect the increased content of TF BS motifs in genomic loci obtained from ChIP- seq massive sequencing data, i. e. increased content of TF BS motifs compared to their expected content due to random reasons. The negative set of DNA sequences is applied to estimate this expected motif content. We have shown that for ChIP-seq peaks, it is more efficient to select random genome loci matching the peaks in G/C-content into the negative set than to use synthetic sequences obtained from the peaks by nucleotide shuffling (Raditsa et al., 2024).

Once enriched BS motifs have been identified for a given ChIP-seq dataset of peaks, the analysis of statistical patterns of motif occurrences in pairs can identify the mechanisms of action of TFs. The concepts of synergy and antagonism of motifs within composite elements (CEs), as stable pairs of motifs, have been previously proposed (Kel et al., 1995). Synergy means that the result of the action of a pair of TFs is notably superior to that of each of them separately. Antagonism, on the contrary, implies that TFs impede each other. For example, one of two TFs is an activator and the other is a repressor, so that one displaces the other. Unfortunately, the concepts of synergy and antagonism refer to a stable pair of two motifs occurring in DNA, and these two cases cannot be distinguished by the frequencies of co-occurrence in the pair of motifs.

More than 15 years have passed since the era of massive sequencing of TF BS began (Jonhson et al., 2007); today, the role of bioinformatics analysis of whole-genome data in understanding the mechanisms of TF’s action cannot be overestimated. In the case of ChIP-seq data, bioinformatics analysis does not deal with individual loci in the genome, but with a set of hundreds or even thousands of such loci where both direct and indirect binding of the target TFs can be observed. In moving from separate consideration of the frequencies of two TF BS motifs in a set of ChIP-seq peaks to observation of statistical patterns in their pairs, it is reasonable to consider two possibilities for these two motifs:

• they co-occur more frequently in the same peaks than it is expected by chance and less frequently occur separately in different peaks;

• they occur more often in different peaks and less often cooccur in the same peaks.

**Fig. 1.**
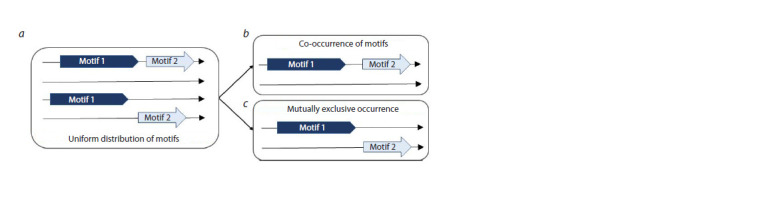
Schema of the distinction between the terms of co-occurrence and mutually exclusive occurrence of TF BS motifs Let the frequency of occurrence of each of the two motifs in a peak be 50 %. a – the two motifs appear in the peaks independently of each other, there are four equally likely cases of motif mapping in the peaks; b – co-occurrence means that both motifs are in the same peak or neither of them is present; c – mutually exclusive occurrence denotes that only one of two motifs can be found in a peak. The arrows from panel a to panels b and c indicate that the four cases of panel a are exactly separated into two groups of two cases in panels b and c.

Therefore, we propose the terms of co-occurrence and mutually exclusive occurrence for the pair of TF BS motifs (Fig. 1).

**Fig. 2.**
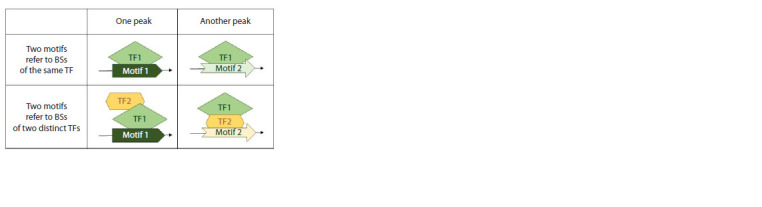
Presumed origin of the mutually exclusive occurrence of two TF BS motifs in a set of ChIP-seq peaks. The two columns represent two different peaks. Mutually exclusive occurrence in a pair of motifs could mean that either the pair of motifs represents two structurally distinct motifs of the same TF (this TF recognize these two motifs in different peaks), or the pair of motifs corresponds to BSs of different TFs. In this case, we assume that one TF interacting directly with DNA is replaced by another TF in some multiprotein complex (TF1 by TF2).

Co-occurrence in a pair of motifs reflects the presence of a CE, a pair of closely located TF BS motifs in DNA, a small spacer between them, or they overlap (Kel et al., 1995; Levitsky et al., 2019). Mutually exclusive occurrence in a pair can have two explanations. Either it represents two structural types of the BS of the same TF (it binds differently in various peaks), or these two BSs belong two distinct TFs. Assuming that the two BS motifs correspond to two distinct TFs within the same multiprotein complex, we can propose that one TF interacting directly with DNA is replaced by another TF. Therefore, the trend of divergence of BS motifs of two TFs into different peaks may indicate a functional relationship of these motifs, in the simplest case representing the aforementioned substitution. For a co-occurrence, in the case of both synergy and antagonism, the two TFs bind to DNA in close proximity to each other (at least for some time they may be in contact even in antagonism), most likely they are within the same multiprotein complex. In the case of mutually exclusive occurrence, on the contrary, the BS motifs and the corresponding TFs are in distant DNA regions (different peaks). Therefore, we assume that the two motifs represent alternative traces of one common molecular function of TFs:

• the same TF recognizes two BS motifs of different structure, or

• binding to DNA occurs through distinct TFs and their BS motifs; these TFs are in the same multiprotein complex. Figure 2 shows both these possibilities.

The AUC ROC (Area Under Curve) is the traditional quantitative measure of the accuracy of a binary classifier. The term ROC stands for Receiver Operating Characteristic curve. For the TF BS motif, the ROC curve is defined as the dependence of the fraction of predicted sequences from the positive set (TPR, True Positive Rate) on the fraction of predicted sequences from the negative set (FPR, False Positive Rate). However, for TF BS motif recognition models in ChIP- seq data, it is more efficient to measure FPR as the expected frequency of a motif in the negative sequence set, but not as the fraction of predicted sequences for this set. This provides higher accuracy of assessment of motif model predictions at stringent and even medium recognition thresholds (Tsukanov et al., 2022). For the TF BS motif recognition model, the recognition accuracy can be calculated as the partial area under the ROC curve (pAUC ROC) (Tsukanov et al., 2022). The pAUC ROC value is equal to the fraction of the area under the curve bounded by the maximum allowable expected frequency of a motif. The area under the ROC curve integrates the fraction of peaks having the predicted TF BSs (the fraction of correctly predicted peaks, Y axis) over a wide range of recognition thresholds, calculated as the frequency of the motif in the negative set (X axis).

In this study, we propose the MetArea approach, which considers two separate “single” motifs as well as a “joint” motif, meaning the occurrence of either of the two single motifs. To predict a joint motif in a DNA sequence, it is sufficient to predict at least one of the two single motifs in it at a given threshold of expected motif frequency. Calculating the frequency of such a joint motif exactly even for a single DNA sequence poses an obstacle due to the huge variety of possible overlaps between single motifs. Therefore, to assess the accuracy of a motif model, we developed and applied the measure of accuracy “Partial area under the PR curve (Precision–Recall)”. To calculate it we need only to track the number of recognized sequences in the positive and negative sets.

The PR curve is the dependence of the Precision measure (the ratio of the number of predicted sequences in the positive set to the number of predicted sequences in the positive and negative sets) on the Recall measure (the ratio of the number of predicted sequences in the positive set to the total number of sequence in this set). The PR curve is an alternative to the more popular ROC curve (Davis, Goadrich, 2006; Keilwagen et al., 2019). The advantage of the area under the PR curve measure over the area under the ROC curve measure is the ratio between the contributions of the mild and stringent recognition thresholds corresponding to the predicted sites of low and high affinity. Compared to the ROC curve, the PR curve provides greater contributions from high-affinity sites than from low-affinity sites. The ROC curve does the opposite. According to the PR curve, the contributions from sites with a low affinity may even tend to zero if such sites do not contain a specific nucleotide context. This is due to equal probabilities of site recognition in the positive and negative sets (Saito, Rehmsmeier, 2015).

We developed the MetArea software package (SP) to identify pairs of TF BS motifs with mutually exclusive occurrence. The MetArea SP calculates the partial area under the PR curve (pAUPRC) accuracy estimates for each of the two input single motifs as well as for their combination, the “joint motif”. This allows the detection of mutually exclusive occurrence of these two input motifs.

## Materials and methods

ChIP-seq data from the GTRD database were used in the analysis (Kolmykov et al., 2021). For each ChIP-seq experiment, a set of 1,000 best quality peaks was analyzed according to preprocessing with the MACS2 tool (Zhang et al., 2008). In this study, enriched motifs obtained from the results of de novo motif search and mouse Mus musculus TF BS motifs from the Hocomoco database (https://hocomoco12.autosome. org/) (Vorontsov et al., 2024) were used in the analyses. De novo search for motifs of the traditional PWM and alternative SiteGA models of TF BS motifs was performed using STREME https://meme-suite.org/meme/tools/streme (Bailey, 2021) and https://github.com/parthian-sterlet/sitega (Tsukanov et al., 2022). The significance of similarity of the enriched motifs from the results of de novo search (STREME motifs) with the motifs of known TFs from the Hocomoco, Cis-BP (Weirauch et al., 2014) and JASPAR (Rauluseviciute et al., 2024) databases was assessed by the TomTom tool https:// meme-suite.org/meme/tools/tomtom (Gupta et al., 2007). The MetArea SP also allows motifs from the Hocomoco and JASPAR databases to be selected for analysis according to the previously used approach (MCOT SP) (Levitsky et al., 2019). The best hit of a motif model has an expected frequency of at least 2E-5 in the set of promoters of all protein-coding genes of the genome. The best hit is given by the predicted site with the highest possible value of the recognition function of a motif model.

In total, the MetArea SP includes 1,420/1,142 motifs for 942/713 human/mouse TFs from the Hocomoco database, and 556/151 motifs for 555/148 plant/insect TFs from the JASPAR database. The MetArea SP is available at https://github.com/ parthian-sterlet/metarea. For a detailed description of the MetArea SP algorithm, see the Results section below. The MetArea SP implements the approach from the MCOT SP (Levitsky et al., 2019) to assess the similarity of the analyzed motifs of the PWM model (nucleotide frequency matrices).

## Results

General description of the MetArea SP

**Fig. 3.**
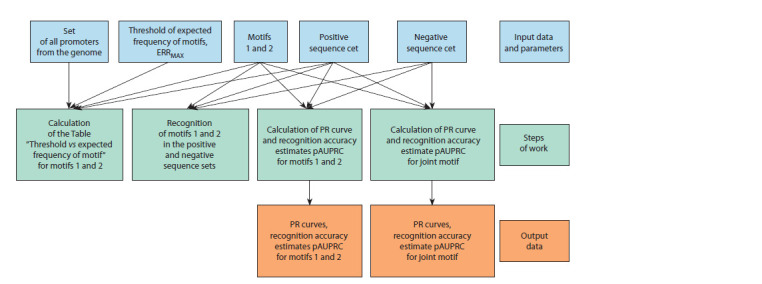
General scheme of the MetArea SP pipeline

The MetArea SP allows analyzing both pairs of motifs of the traditional PWM model and pairs of motives of the traditional PWM and alternative SiteGA models (Levitsky et al., 2007; Tsukanov et al., 2022). Figure 3 presents the general scheme of the MetArea SP pipeline.

The input data and parameters of the MetArea SP are listed below:

• Two motifs: (1) a combination of two motifs of the PWM model given by two nucleotide frequency matrices (NFMs), or (2) a combination of a motif of the PWM model given by an NFM and a motif of the SiteGA model given by its weight matrix, see https://github.com/parthian-sterlet/sitega (Tsukanov et al., 2022).

• Positive set in FASTA format (the set of ChIP-seq peaks, NF sequences, Number of Foreground sequences).

• Negative set in FASTA format (NB sequences, Number of Background sequences); it is recommended to prepare it in advance from the positive set and the whole genome by the AntiNoise SP (Raditsa et al., 2024), https://github.com/ parthian-sterlet/antinoise. For each sequence of the positive set, several sequences of the negative set are selected randomly in the whole genome by its length and G/C-content. Further in the analysis, NF/NB = 5.

• The set of promoters of all genes of the genome is required to determine recognition thresholds based on the calculation of the Table ‘Threshold vs. ERR’ (“Recognition function threshold vs. Motif frequency in the set of all genome promoters”) for each of the input motifs

• The ERRMAX threshold for the maximum expected motif frequency (Expected Recognition Rate, ERR) for each input motif.

• Tables ‘Threshold vs. ERR’ for each input motif.

The maximum motif frequency of 0.01 means that BS specificity corresponds to one site per one hundred nucleotide positions. The recommended range for the threshold of expected motif frequency ERRMAX is 0.001 to 0.01. The ERRMAX value of 0.002 is used below. We have previously used the ‘Threshold vs. ERR’ tables to set recognition thresholds across motifs (Levitsky et al., 2019; Tsukanov et al., 2021, 2022). Each motif and its ‘Threshold vs. ERR’ table are presented in a binary-format file generated by the MetArea SP components to calculate the expected motif frequencies for the PWM and SiteGA motif models.

The outputs of the MetArea SP are:

• A text file with PR curves for each of the input motifs as well as their joint motif

• A text file with the values of pAUPRC recognition accuracy estimates for each of the input motifs, as well as for their joint motif, the value of the ratio of areas under the curves (see below), and the estimate of motifs’ similarity (for pairs of PWM motifs only).

Definition of recognition thresholds for different motifs

The recognition function thresholds of each of the two input motifs, according to pre-calculated ‘Threshold vs. ERR’ tables, are transformed into a common scale of expected motif frequency, ERR (Levitsky et al., 2019; Tsukanov et al., 2021, 2022). This is necessary to construct the PR curve of the joint motif. The expected motif frequency ERR for the input motifs is calculated up to the threshold ERRMAX, so that all expected frequencies satisfy the criterion: ERR < ERRMAX.

The expected motif frequency in the promoter set was calculated as follows. The values of the motif recognition function for each predicted site in the set at each position and DNA strand were determined. Then, for each recognition threshold, the expected motif frequency was calculated as the ratio of the number of predicted BSs with the recognition function values equal to or higher than the recognition threshold to the total number of positions available for such BSs in the set in both DNA strands.

Statistical metrics and the PR curve

The PR curve (Davis, Goadrich, 2006) for the TF BS motif model can be defined as follows: the X axis means the ratio of the number of sequences from the positive set (peaks) with predicted sites to the number of all peaks (TPR, True Positive Rate, Recall, REC):

**Formula. 1.**
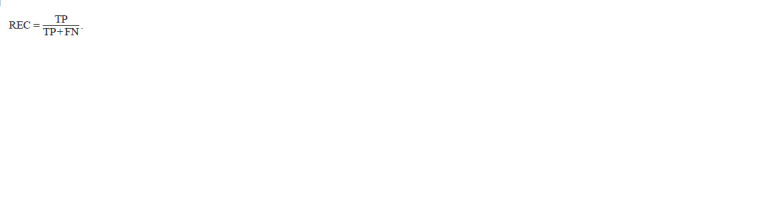
Formula1

Here, TP/FN (True Positives/False Negatives) is the number of correctly/incorrectly predicted sequences from the positive set (TP + FN = NF).

The Y axis of the PR curve implies the ratio of the number of predicted sequences in the positive set to the number of all predicted sequences in positive and negative sets (Precision, PREC), according to (Davis, Goadrich, 2006):

**Formula. 2.**
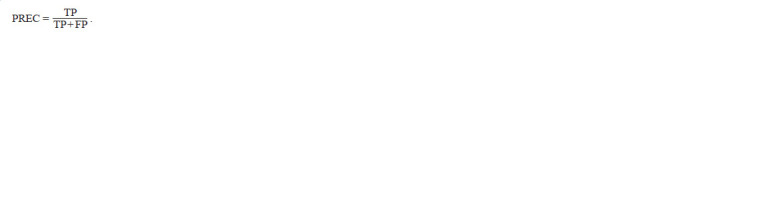
Formula2

Here, FP (False Positives) is the number of predicted sequences in the negative set. Taking into account the difference in the number of sequences between the positive (NF) and negative (NB) sets, we corrected the calculation of the Precision value as follows:

**Formula. 3.**
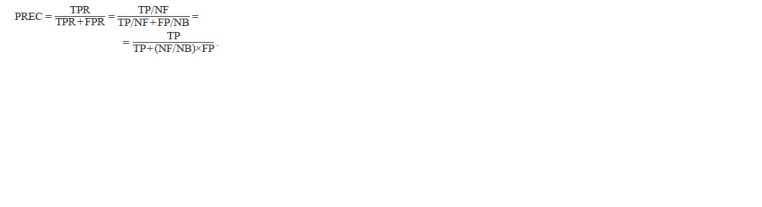
Formula3

Here, TPR and FPR are the fractions of predicted sequences in the positive and negative sets. The NF/NB coefficient takes into account the difference between the sizes of negative (NB) and positive (NF) sets. The expected numbers of predicted sequences of positive (TP) and negative (FP) sets due to random reasons are proportional to the set sizes, NF and NB, respectively. Hence, we introduce the NF/NB coefficient to unify the behavior of the PR curve for different ratios of positive and negative set sizes.

Partial area under the PR curve and the ratio of areas under curves

**Fig. 4.**
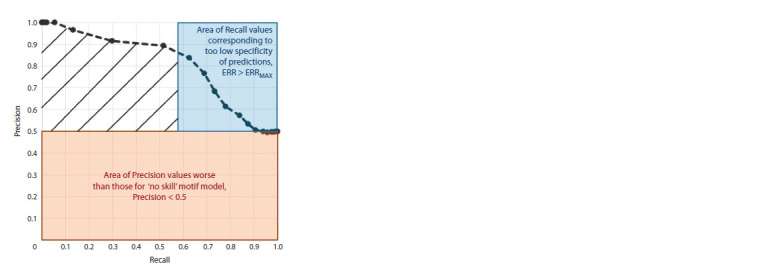
Scheme of calculation of the partial area under the PR curve. The X axis is the Recall measure (the probability of predicting the positive set sequence, Recall = TPR = TP/NF), formula (1). The Y axis is the Precision measure, the ratio of the probability of predicting the positive set sequence to the sum of the probabilities of predicting the positive and negative set sequences, Precision = TPR / (TPR + FPR), formula (3). The pink area marks Precision < 0.5 values corresponding to predictions worse than those of a “no skill” model equally likely to predict sequences in the positive and negative sets. The criteria Precision > 0.5/Precision < 0.5 mark areas of selection towards the positive/ negative sets. The blue area shows the area of predicted sequences of the positive set with very low specificity. They correspond to the expected frequency of the motif greater than the threshold, ERR > ERRMAX. The normal distribution with the mean and standard deviation (μN, σN) = (5, 2.5) was taken to generate the data of the negative set example, and the positive set was a mixture of 50 %/50 % normal distributions (μP1, σP1) = (10, 1) and (μP2, σP2) = (5.5, 4). These distributions model sites passing and failing to pass the threshold ERRMAX of the expected motif frequency. The shading denotes the area determining the metric pAUPRC as the partial area under the curve.

The MetArea algorithm uses the tables “Recognition function threshold vs. Motif frequency in the set of all genome promoters” described above, and performs recognition of two input single motifs in the positive and negative sets. Next, the pAUPRC measure is calculated for the single motifs as well as for the joint motif. The calculation of the partial area under the curve PR (pAUPRC) is limited by the criteria imposed on the Recall (X axis) and Precision (Y axis) measures, that is, the area is partial on both the X axis and the Y axis. The example in Figure 4 explains the choice of the partial area in both axes.

The criterion for the partial area under the PR curve on the X axis is the participation in the calculation of the pAUPRC measure of a part of the whole range of the Recall measure from 0 to 1. This criterion means that not all peaks with predicted sites are involved, but only those peaks, the best hits of which have an expected frequency below the threshold, ERR < ERRMAX (Fig. 4). Here, we chose the milder threshold of the expected frequency (ERRMAX = 0.002) than the one previously used to analyze the motifs of target TFs (ERRMAX = 0.001) (Tsukanov et al., 2022). We previously analyzed the motifs of target TFs of ChIP-seq experiments, and the MetArea SP analyzes the BS motifs of both target TFs and less conservative ones of partner TFs.

The criterion for the partial area under the PR curve on the Y axis subtracts from each value of the Precision measure its expected value PRECEXP (Fig. 4) (Saito, Rehmsmeier, 2015). For a model that is equally likely to recognize sequences from the positive and negative set, the PR curve is a horizontal line:

**Formula. 4.**
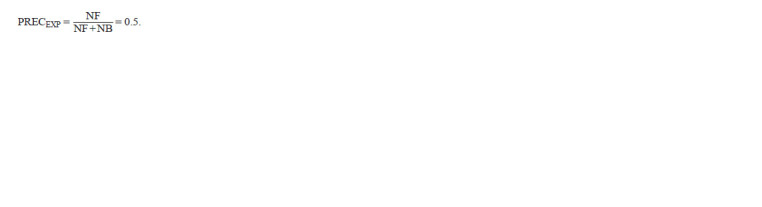
Formula4

This ratio is constant and equal to 0.5 because the FP value was normalized above, so the set sizes in this formula can already be considered equal. Hence, the partial area under the PR curve in the MetArea SP is calculated as the following sum:

**Formula. 5.**
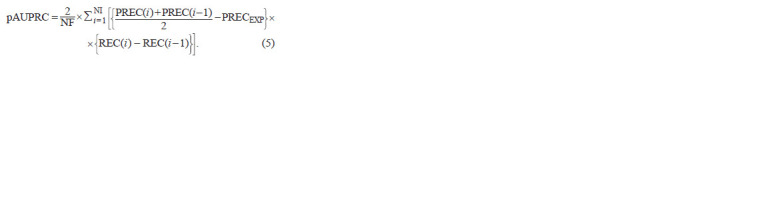
Formula5

Here, NI is the mildest threshold, determined as described above from the expected frequencies and the input parameter ERRMAX. The 2/NF factor is required to normalize the value of pAUPRC to the maximum value of 1. The maximum value of the first multiplier under the sum, {(PREC(i ) + PREC(i – 1))/2 – – PRECEXP}, is 0.5 since the maximum Precision value is 1; and the maximum value of the sums of the second multipliers, {REC(i ) – REC(i – 1)}, is NF, the size of the positive set.

The criterion for predicting the functional relation of motifs reflects the increase in the accuracy estimate of the joint motif compared to the accuracy estimates of single motifs. This criterion quantitatively assesses mutually exclusive occurrence in pairs of motifs. For a pair of motifs A and B, the criterion requires a higher value of the accuracy estimate pAUPRC(A&B) of the joint motif A&B compared to the values of the accuracy estimates of both single motifs, pAUPRC(A) and pAUPRC(B). Calculated as follows, the Ratio of Areas Under Curves (RAUC) should exceed one:

**Formula. 6.**
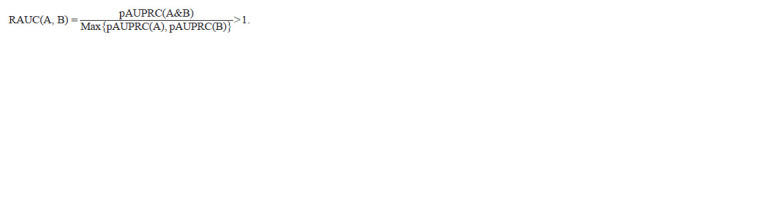
Formula6

Application options of the MetArea SP

MetArea SP inputs can be TF BS motifs with expected enrichment in the positive vs negative set, e. g., such motifs are the results of a de novo motif search (Bailey, 2021). Separate applications of SP implement massive analyses of the collections of TF BS motifs from the Hocomoco and JASPAR databases. Analysis of multiple pairs of motifs allows identification of pairs that reveal a larger increase in pAUPRC recognition accuracy estimates when motifs are combined. The MetArea SP allows several application options, implemented as separate programs. The following application options consider the PWM motif model:

• two given motifs;

• several given motifs, for K motifs all possible {K × (K – 1)/2} pairs are checked;

• a given motif vs all M motifs of BS of known TFs from the database. For a given motif, all its M pairs with the motifs from the Hocomoco (human, mouse) or JASPAR (plants, insects) collections are checked;

• all BS motifs of known TFs from the database are checked. From all M motifs of known TFs from the Hocomoco or JASPAR collection, K motifs with the highest pAUPRC accuracy scores are selected and all {K × (K – 1)/2} possible pairs of these motifs are tested. The application options for the PWM and SiteGA motif models:

models: • motif PWM and motif SiteGA.

Next, we provide examples of the results of ChIP-seq data analysis for different application options of the MetArea SP.

Analysis of several given motifs of the PWM model

**Fig. 5.**
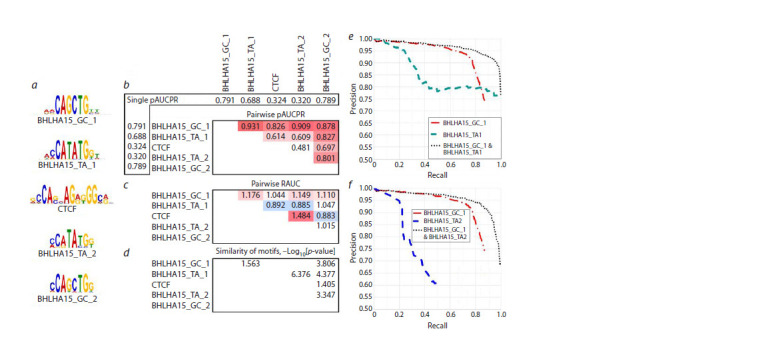
Analysis of the five most enriched motifs from the de novo motif search results (STREME) (Bailey, 2021) for the ChIP-seq dataset for mouse BHLHA15 TF (Hess et al., 2016) (GTRD PEAKS039234, GEO GSM2299654/GSM2299655). a – sequence logos for five motifs, sorted by enrichment significance obtained from the STREME tool; BHLHA15 TF BS motifs are labelled according to the dinucleotide in their spacer in the CAnnTG consensus; b – table of pairwise values of pAUPRC accuracy estimates of the joint motifs constructed from pairwise combinations of motifs, headers indicate pAUPRC values for single motifs, shades of red mark the maximum pAUPRC values of the joint motifs; c – table of pAUPRC values in pairs of motifs, shades of red and blue mark values greater than and less than one; d – table of significances of motifs similarity, –Log10[p-value]; e, f – PR curves for single motifs and their pairwise joint motifs BHLHA15_GC_1/BHLHA15_TA1 and BHLHA15_GC_1/BHLHA15_TA2.

Consider the ChIP-seq dataset for the BHLHA15 TF (Hess et al., 2016) (GTRD PEAKS039234, GEO GSE86289) for mouse pancreas. Application of a de novo search (STREME tool) (Bailey, 2021) showed that among the five motifs with the highest enrichment, four had significant similarity ( p < 0.001) (Gupta et al., 2007) to known BHLHA15 TF BS motifs from the Hocomoco. The motifs #1/#5 and #2/#4 are similar to BHA15.H12CORE.0.P.B and BHA15.H12CORE.1.SM.B, respectively (Fig. 5а). These motifs correspond to the consensus E-box CAnnTG with spacers GC and AT, so they are labelled BHLHA15_GC_1/BHLHA15_GC_2, and BHLHA15_AT_1/BHLHA15_AT_2, respectively. Motif #3 has significant similarity ( p < 0.001) to the BS motif of the CTCF TF (CTCF. H12CORE.0.P.B) (Fig. 5а).

Analysis of the values of the pAUPRC recognition accuracy estimates for single motifs and their pairwise joint motifs (Fig. 5b) is based on the corresponding RAUC values for pairs of motifs (Fig. 5c), the similarity assessment of pairs of motifs is required to control for significantly similar motifs (Fig. 5d ). High RAUCs are found for the pairs of motifs BHLHA15_ GC_1/BHLHA15_TA2 and BHLHA15_ GC_1/ BHLHA15_TA1, the PR curves for them are shown in Figure 5e, f. The CTCF motif has high RAUCs with BHLHA15_ GC1 and BHLHA15_TA2 motifs (Fig. 5c). The pair of BHLHA15_TA2 and CTCF motifs was found to have the maximum RAUC of 1.48 (Fig. 5c). Overall, our results are consistent with the ability of the TF BHLHA15 to bind to DNA only as part of the dimer of two bHLH TFs (Amoutzias et al., 2008). The trend towards divergence of BSs of various structure of the BHLHA15 TF into different peaks could mean that (1) the dimer may comprise different TFs from the bHLH class (including BHLHA15 TF), and (2) the binding of the dimer is influenced by other partner TFs, that form multiprotein complexes with the BHLHA15 TF. Hence, the DBD of the BHLHA15 TF adopts various conformations, e. g., TF CTCF, the BS motif of which is also enriched (Fig. 5а), may be a partner TF. According to experimental data: (1) several TFs from the bHLH class have protein-protein interactions with the CTCF TF (BIOGRID database, https:// thebiogrid.org/); (2) analysis of partner TFs by genomic colocalization (Hu et al., 2020) confirms that several TFs from the bHLH class are co-localized with CTCF TFs at the same genomic loci in vivo.

Analysis of all BS motifs of known TFs from the database

**Fig. 6.**
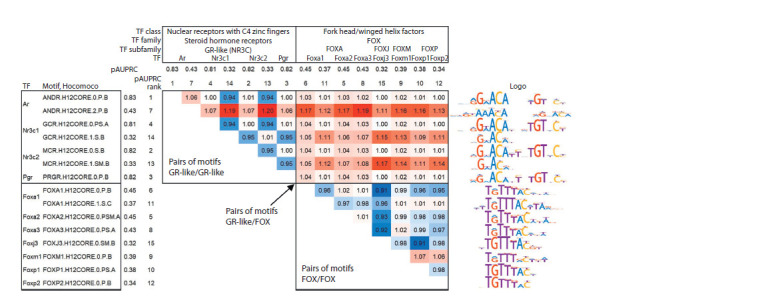
Results of the analysis of BS motifs of known TFs from the Hocomoco database for the ChIP-seq dataset for AR TF in mouse prostate (Chen et al., 2013). The 15 most enriched motifs according to the pAUPRC accuracy estimates are included in the analysis, headers of rows and columns show values and ranks of the pAUPRC metrics and the names of TFs from the Hocomoco database. Row headers indicate motif identifiers from Hocomoco, and column headers indicate the names of the TF class, family, and subfamily. In the table, shades of red/blue indicate changes in RAUC up/down from the neutral value of 1. The rightmost column shows the sequence logos of the motifs from the Hocomoco database. Black rectangles mark GR-like and FOX motifs in row and column headers, and in the table, pairs of BS TF motifs GR-like/GR-like, GR-like/FOX and FOX/FOX.

Consider the ChIP-seq dataset for TF AR (Androgene Receptor) for the mouse prostate (Chen et al., 2013) (GTRD PEAKS035588, GEO GSM1145307). Figure 6 for this ChIP-seq dataset shows the matrix of the pairwise RAUC values for the 15 most enriched TF BS motifs according to the pAUPRC measure out of all 1,142 mouse TF BS motifs from the Hocomoco database. Among these 15 motifs, seven motifs belong to the TF AR BS and its homologues from the same subfamily GR-like (NR3C) {2.1.1.1.1} of the Steroid hormone receptors {2.1.1} family of the Nuclear receptors with C4 zinc fingers {2.1} class. This family defines the target TF AR, and the likely motifs of its BS. The other eight motifs out of 15 belong to BS of TFs from the subfamilies FOXA {3.3.1.1}, FOXJ {3.3.1.10}, FOXM {3.3.1.13} and FOXP {3.3.1.16}. They comprise the same FOX family {3.3.1} from the class Fork head/winged helix factors {3.3}. TFs of this family are putative partner TFs for AR TFs, e. g. Foxa1 TF is known for the same prostate tissue (Yang, Yu, 2015).

The pAUPRC values are greater than 1 for almost all pairs of GR-like/FOX motifs. For example, the RAUC value of 1.03 for the ANDR.H12CORE.0.P.B (pAUPRC rank 1) and FOXA2.H12CORE.0.PSM.A (rank 5) pair corresponds to the maximum value pAUPRC = 0.853 among all pairs of GR-like/ FOX motifs. The pAUPRC values for pairs of GR-like/ GR-like motifs exceed the value of 1 only for some pairs of motifs. The ANDR.H12CORE.2.P.B motif (rank 7) has a distinct consensus among all other GR-like motifs (AAACA instead of GNACA, see the Logo column, Figure 6); it has high RAUC values, and this is the only motif with RAUC values above 1 in all pairs with other GR-like and FOX motifs. In particular, among pairs of GR-like/GR-like motifs, the maximum pAUPRC value of 0.876 with a RAUC of 1.06 is achieved for the pair of motifs ANDR.H12CORE.0.P.B (rank 1) and ANDR.H12CORE.2.P.B (rank 7). Also, high RAUC values in pairs of GR-like/GR-like motifs were found for the MCR.H12CORE.1.SM.B motif, but it has the lowest pAUPRC rank of 15. This motif is a monomer-binding motif, not a dimer. Among the FOX/FOX motif pairs, there are almost no RAUC values greater than 1.

Overall, the high RAUC values of many pairs of GR-like/ GR-like motifs suggest that the AR TF binds in different peaks using distinct structural types of GR-like motifs. A similar assumption can be made on the binding of a TF dimer consisting of AR and a TF from the FOX family according to the high RAUC values for pairs of GR-like/FOX motifs. The results obtained for ChIP-seq data for the AR TF imply the following. (1) Binding of AR TF to DNA occurs in the AR/AR and AR/ Foxa1 dimers (if it is the Foxa1 TF that binds to FOX motifs under experimental conditions), and (2) both TFs allow a large variety of different structural types of BSs, so various pairs of motifs diverge in different peaks.

Analysis of the pair of motifs of the PWM and SiteGA models

**Fig. 7.**
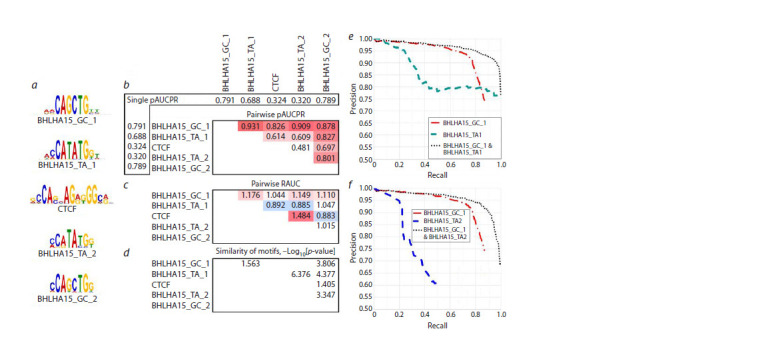
Results of analysis of the motif pair of the PWM and SiteGA models by the MetArea SP. Red, blue, and black represent PR curves for the PWM, SiteGA motifs, and the joint motif PWM & SiteGA. The ChIP-seq dataset for TF E2F4 (GTRD PEAKS035857, GEO GSM881061) was used in the analysis.

Consider the ChIP-seq dataset for the E2F4 TF for primary innate immunity dendritic cells derived from mouse bone marrow stimulated with the pathogenic component lipopolysaccharide for 120 minutes (Garber et al., 2012) (GTRD PEAKS035857, GEO GSM881061). Figure 7 shows the PR curves for the PWM, SiteGA, and their joint PWM & SiteGA motifs calculated by the MetArea SP. The pAUPRC values for the PWM, SiteGA, and the joint PWM & SiteGA motifs are 0.457, 0.358, and 0.47, respectively; the pAUPRC value of the joint motif is 1.028.

The PWM and SiteGA motif models are based on very different methodological principles (Levitsky et al., 2007). The PWM model represents high-affinity sites defined by the most conserved positions and the most frequent nucleotides in them. The SiteGA model comprises sites containing dependencies of different positions that presumably originate from the common actions of at least two TFs in cooperative binding to DNA (Morgunova, Taipale, 2017; Levitsky et al., 2020). Predicted sites of the SiteGA model are markedly less conserved than those of the PWM model; the SiteGA model is able to predict low affinity sites better than the PWM model (Tsukanov et al., 2022). Combining the PWM and SiteGA models improves recognition of low-affinity sites, as reflected by the greater extent of the PR curve of the joint PWM& SiteGA motif on the X axis (Recall), compared to each of the single PWM and SiteGA motifs. Although the joint motif has smaller Precision values (Fig. 7, Y axis) than the PWM model, the wider range of Recall values (X axis) determines the increase in the pAUPRC measure of the joint motif. Single motifs up to the threshold of expected motif frequency ERRMAX = 0.002 recognize 73.2 % (PWM) and 63.3 % (SiteGA) of peaks, the joint motif recognizes 79.9 %.

The hypothesis that the PWM and SiteGA models represent different structural types of the E2F4 TF BS is confirmed by the TomTom motif comparison tool ( p-value < 0.05) (Gupta et al., 2007). To prove this, for the PWM model, we used its nucleotide frequency matrix, and for the SiteGA model, as previously (Tsukanov et al., 2022), the nucleotide frequency matrix constructed from the predicted sites. The ability of the E2F4 TF to bind to different structural types of BSs is also indicated by the experiment of M. Garber et al. (2012), where the genomic binding loci of 25 TFs were determined under the same conditions. The loci of E2F4 TFs were shown to overlap significantly with the loci of five TFs: EGR2, EGR1, IRF2, ETS2 and E2F1. Consequently, it can be assumed that the TF E2F4 is part of the same multiprotein complexes with these TFs. Therefore, in different TF loci, E2F4 has to change its BSs to a greater or lesser extent to adapt to the BSs of partner TFs.

## Discussion

In our study, we propose the novel MetArea approach for detecting mutually exclusive occurrence in pairs of TF BS motifs based on analyses of single ChIP-seq datasets. If two motifs are structurally distinct BS motifs of the same TF in various peaks, then the mutually exclusive occurrence is due to the preferences of this TF to bind to either one or the other structural type of BS in the peaks, but it is less common to observe two BSs of different structures in the same peaks. If the BS motifs belong to two different TFs, mutually exclusive occurrence can result from the participation of both TFs in the same multiprotein complexes, but in different peaks one or another TF binds to DNA directly, but it is less common to observe BSs of both TFs in the same peak.

During the development of the MetArea SP, we abandoned the use of the metric of the partial area under the ROC curve (pAUC ROC) (Levitsky, Tsukanov, 2024) and used the metric of the area under the PR curve (Davis, Goadrich, 2006) to determine the metric of the partial area under the PR curve. It had been previously proposed (Davis, Goadrich, 2006) that the application of the area under the AUC ROC curve cannot be correct if the actual recognition thresholds of a binary classifier should be quite stringent. Therefore, we should take note if the advantage of one motif relative to another is recruited in the interval of mild recognition thresholds (at the right tail of the ROC curve). To correctly compare two motifs in this case, instead of the metric of the area under the AUC ROC curve, we previously used the metric “Partial Area Under the ROC Curve, pAUC”. Instead of the full-size range of the False Positive Rate (FPR, the fraction of recognized objects from the negative set, X axis of the ROC curve) from 0 to 1, this metric uses only a certain left part of it, discarding the range of too large FPR values. We implemented this approach to compare the recognition accuracy of TF BS motifs of the PWM, BaMM and SiteGA models (Tsukanov et al., 2022). There, we used the criterion on the Expected Recognition Rate, ERR <0.001, to restrict the recognition thresholds of motifs in order to compute the pAUC ROC accuracy estimates.

Unfortunately, this approach is not suitable to compute the accuracy of the joint motif required in the implementation of the MetArea approach. The rationale for this is the necessity to count the frequency of the joint motif, i. e. the number of its hits. It is possible for non-overlapping single motifs, and in the case of their overlapping, the frequency of the joint motif should be reduced in some way. An alternative way to get rid of the overestimation of accuracy given by the AUC ROC measure is to switch from the ROC curve to the PR curve and calculate the area under the PR curve (Davis, Goadrich, 2006; Keilwagen, et al., 2019).

Several approaches have been previously proposed to identify the occurrence of different TF BS motifs or different sets of motifs in various peak fractions of a single set of ChIP-seq peaks. The DIVERSITY tool (Mitra et al., 2018) partitions a set of ChIP-seq peaks into several non-overlapping groups, so that each group is represented by its enriched motif from de novo search results. Later, the authors allowed that each group of peaks is not represented by a single motif, but by a combination of several motifs. The cisDIVERSITY tool (Biswas, Narlikar, 2021) for the set of peaks performs a de novo search for enriched motifs using the PWM model, and then distributes the found motifs into several non-overlapping groups of peaks so that all groups make up the entire set of peaks. Each of the motifs has different frequencies across groups, e. g., some groups have higher frequencies than other groups, while other groups may not have a motif. The tasks of the DIVERSITY/cisDIVERSITY and MetArea tools are similar in that different motifs are separated into certain fractions of peaks. However, the DIVERSITY/cisDIVERSITY tools: (1) identify the entire variety of motifs and divide all peaks into groups in order to find distinct motifs or combinations of them for different groups; (2) consider only the traditional PWM motif model. The MetArea SP (1) considers only pairs of motifs, to find pairs of motifs that better complement each other by maximizing the accuracy measure pAUPRC for the joint motif; (2) considers both the traditional PWM model and alternative models of the TF BS motif.

## Conclusion

We have developed the MetArea SP. It uses a single set of ChIP-seq peaks to calculate the “Partial Area Under the PR Curve” (pAUPRC) accuracy measure for the two input single TF BS motifs, determines the joint motif from them, and also calculates the pAUPRC measure for it. Creating a joint motif from the two single motifs and calculating a pAUPRC accuracy estimate for it allows comparing two single motifs and their overall effect on a uniform scale. The excess of accuracy estimates of the joint motif over those of both single motifs indicates their mutually exclusive occurrence. The results of the MetArea analysis allow predicting the functional relationship of the two motifs, and hence their corresponding TFs. In particular, the MetArea SP can offer substantial arguments for or against the hypothesis that the two motifs are structural types of the BS of a single TF. Similarly, support or rejection are proposed for the hypothesis that the BS motifs represent two TFs together involved in the regulation of gene transcription as part of a single multiprotein complex. In summary, the MetArea SP predicts for a given ChIP-seq dataset (1) structural diversity of BSs of a single TF and (2) pairs of BS motifs of different TFs acting to regulate gene transcription as part of single multiprotein complexes of many TFs.

## Conflict of interest

The authors declare no conflict of interest.
